# Items of General Interest

**Published:** 1916-06

**Authors:** 


### Items of General Interest.

The United States Civil Service Commission announces an open competitive examination for clinical director, for men only. From the register of eligibles resulting from this examination certification will be made to fill a vacancy in this position in the government hospital for the insane at Washington, D. C., at a salary of $2000 per annum with maintenance in the hospital, and vacancies as they may occur in positions requiring similar qualifications, unless it is found to be in the interest of the service to fill any vacancy by reinstatement, transfer or promotion. Application should be properly executed, excluding the medical and county officer's certificates, and must be filed with the commission at Washington prior to the hour of closing business on June 27, 1916.

Dr. E. H. Cary, of Dallas, was chosen President-elect and will automatically become President at the close of the convention in Dallas next year. The Association is to be congratulated for honoring with this high office a man so capable, who has stood for many years in the front ranks of progressive medicine—a leader among leaders in medical activities.

Dr. Cary has been interested in medical college affairs for many years, and it is through his influence that the Baylor Medical College has been brought so prominently to the front.

It was under the presidency of Dr. Cary that the Dallas County Medical Society first began its really active work in Dallas.

---

Miss Jessie Gillender, who died in Los Angeles recently, bequeathed $100,000 to the medical school of Johns Hopkins University for the establishment of the Epilepsy Medical Research Fund. The income of the fund is to be used for investigation into the cause, prevention and cure of epilepsy.

---

In a bulletin which the committee has just issued and addressed to the mayors, health officers, editors, citizens and taxpayers is given the statistical results of its investigation. The committee's questionnaire was sent to the health officers of 252 cities. Infant mortality statistics were obtained for 144 of these. From the remaining 108 either no reliable data could be obtained or the health officials are unwilling to supply the information which the committee requested. Out of the 144 cities furnishing information, 46 were cities of 100,000 or more population, 32 were cities of 50,000 to 100,000 population and 66 were cities of 15,000 to 50,000 population, according to the last U. S. census.

Of the cities with a population of 100,000 or more a baby born in Omaha, Nebraska, was found to have four times as good a chance to live to celebrate its first anniversary of its birth as a baby born in Nashville, Tennessee, or Fall River, Massachusetts. In the cities under 100,000 and over 50,000 population, a Salt Lake City baby has over three times the chance to survive the first year of life as a Passaic, N. Y., or Holyoke, Mass., baby has, while in cities between 25,000 and 50,000 population a La Crosse, Wis., baby has an advantage of more than six to one over a Montgomery, Ala., or Perth Amboy, N. J., baby.

Mr. Taylor, the Director of the New York Milk Committee, maintains that it is the community rather than the individual that has the power to reflect the ultimate credit on itself and its individuals in baby saving as in everything else. He states no community can excuse a high infant death rate on the ground that conditions are unfavorable. We now know that conditions which endanger the health and lives of babies can be corrected if only the known preventive measure is applied.

### How the Government is Meeting the Malaria Problem.

Four per cent of the inhabitants of certain sections of the South have malaria. This estimate, based on the reporting of 204,881 cases during 1914, has led the United States Public Health Service to give increased attention to the malaria problem, according to the annual report of the Surgeon General. Of 13,526 blood specimens examined by government officers during the year, 1797 showed malarial infection. The infection rate among white persons was above 8 per cent, and among colored persons 20 per cent. In two counties in the Yazoo Valley, 40 out of every 100 inhabitants presented evidence of the disease.

Striking as the above figures are they are no more remarkable than those relating to the reduction in the incidents of the disease following surveys of the Public Health Service at thirty-four places in nearly every State of the South. In some instances from an incidence of 15 per cent, in 1914, a reduction has been accomplished to less than 4 or 5 per cent, in 1915.

One of the important scientific discoveries made during the year was in regard to the continuance of the disease from season to season. Over 2000 Anopheline mosquitoes in malarious districts were dissected, during the early spring months, without finding a single infected insect, and not until May 15, 1915, was the first parasite in the body of a mosquito discovered. The Public Health Service, therefore, concludes that mosquitoes in the latitude of the Southern States ordinarily do not carry the infection through the winter. This discovery indicates that protection from malaria may be secured by treating human carriers with quinine previous to the middle of May, thus preventing any infection from chronic sufferers reaching mosquitoes and being transmitetd by them to other persons.

Although quinine remains the best means of treating malaria and is also of marked benefit in preventing infection, the eradication of the disease as a whole rests upon the destruction of the breeding places of Anopheline mosquitoes. The Public Health

Service, therefore, is urging a definite campaign of draining standing water, the filling of low places, and the regrading and training of streams where malarial mosquitoes breed. The oiling of breeding places, and the stocking of streams with top-feeding minnows, are further recommended. The Service also gives advice regarding screening, and other preventive measures as a part of the educational campaigns conducted in sections of infected territory.

This study is typical of the scientific investigations which are being carried out by the Public Health Service, all of which have a direct bearing on eradicating the disease. The malaria work now includes the collection of morbidity data, malaria surveys, demonstration work, scientific field and laboratory studies, educational campaigns, and special studies of impounded water and drainage projects.—*United States Public Health Service.*

---

The Roentgen Ray Society elected Dr. W. S. Hamilton, of San Antonio, as President; Dr. B. T. Van Zandt, of Houston, as Vice-President, and Dr. J. W. Torbett, Secretary and Treasurer.

The Abbott Laboratories have issued a most attractive and instructive booklet on "The Therapeutic Uses of the Bulgarian Bacillus" (Galactenzyme). A request from you will bring you the booklet free of charge.

---

Dr. E. W. Stromberg, of Taylor, was recently appointed health officer for that place for the ensuing year.

---

The residence and most of the contents of Dr. and Mrs. J. D. Howard, of Glifton, were destroyed by fire May 10th.

---

The contract has been let for the erection of a three-story rock building to be used as a sanitarium by the Belton Sanitarium Company, recently organized there. It is expected to have the building completed in six weeks.

---

Headed by Dr. John R. McDill, of Milwaukee, forty leading American surgeons sailed for Europe on May 27th to establish in Germany and Austria two field hospitals under the auspices of the American Expeditionary Committee.

---

Dr. E. H. Cary, of Dallas, was chosen President-elect of the Texas State Medical Association at the concluding business session of the House of Delegates.

Dallas was unanimously selected as the meeting place for the 1917 convention of the Association, which convenes in May.

Other officers were elected as follows: Dr. R. Y. Lacy, First Vice-President, Pittsburg; Dr. Chas. R. Johnson, Second Vice-President, Gainesville; Dr. W. L. Brown, Third Vice-President, El Paso; Dr. Holman Taylor, re-elected Secretary, to serve third term of three years, Fort Worth; Dr. W. L. Allison, Treasurer, re-elected, Fort Worth; Dr. John T. Moore, re-elected Chairman of Board of Trustees, to serve five years, Houston; Dr. W. A. King, re-elected member of the Council on Medical Defense, San Antonio; Dr. C. R. Hartsook, re-elected member Board of Councilors from Third District, Wichita Falls; Dr. Chas. S. Venable, member Board of Councilors from Fifth District, San Antonio; Dr. W. N. Wardlaw, re-elected member Board of Councilors from Third District, Corpus Christi; Dr. A. C. Scott, re-elected member Board of Councilors from Twelfth District, Temple; Dr. C. E. Seals, member Board of Councilors from Third District, Daingerfield.

Two delegates to the American Medical Association were chosen by acclamation. Dr. G. H. Moody, of San Antonio, the retiring President, and Dr. Marvin L. Graves, of Galveston, re-elected, were chosen without a dissenting vote. Dr. C. M. Rosser, of Dallas, and Dr. Bacon Saunders, of Fort Worth, were chosen as alternates.

Dr. Ben H. Turner, of Cleburne, was reappointed by the President to serve a term of three years on the Council of Legislation and Public Instruction.

Dr. J. M. Inge, of Denton, President-elect during the year just ended, automatically became President at the conclusion of the convention.

The surgeon general of the army announces that preliminary examinations for the appointment of first lieutenant in the army medical corps will be held on July 17, 1916, and August 14, 1916, at points to be hereafter designated.

Full information concerning these examinations can be procured upon application to the "Surgeon General, U. S. Army, Washington, D. C."

In order to perfect all necessary arrangements for the examination, applications must be completed and in possession of the Adjutant General at least three weeks before the date of examination. There will be more than one hundred vacancies to be filled after July 1st, when the bill for the reorganization of the army becomes a law.

---

Owing to the withdrawal of troops from their regular stations for duty on the Mexican border, the War Department has been compelled to abandon the camps of instruction for officers of the medical reserve corps that were to be held during the coming summer.

